# Are big data analytics helpful in caring for multimorbid patients in general practice? - A scoping review

**DOI:** 10.1186/s12875-019-0928-5

**Published:** 2019-02-27

**Authors:** Alexander Waschkau, Denise Wilfling, Jost Steinhäuser

**Affiliations:** Institute for Family Medicine, University Medical Center Schleswig-Holstein, Campus Lübeck, Ratzeburger Allee 160, Haus 50, 23538 Lübeck, Germany

**Keywords:** Big data analytics, General practice, Multimorbidity, eHealth

## Abstract

**Background:**

The treatment of multimorbid patients is one crucial task in general practice as multimorbidity is highly prevalent in this setting. However, there is little evidence how to treat these patients and consequently there are but a few guidelines that focus primarily on multimorbidity. Big data analytics are defined as a method that obtains results for high volume data with high variety generated at high velocity. Yet, the explanatory power of these results is not completely understood. Nevertheless, addressing multimorbidity as a complex condition might be a promising field for big data analytics.

The aim of this scoping review was to evaluate whether applying big data analytics on patient data does already contribute to the treatment of multimorbid patients in general practice.

**Methods:**

In January 2018, a review searching the databases PubMed, The Cochrane Library, and Web of Science, using defined search terms for “big data analytics” and “multimorbidity”, supplemented by a search of grey literature with Google Scholar, was conducted. Studies were not filtered by type of study, publication year or language. Validity of studies was evaluated independently by two researchers.

**Results:**

In total, 2392 records were identified for screening. After title and abstract screening, six articles were included in the full-text analysis. Of those articles, one reported on a model generated with big data techniques to help caring for one group of multimorbid patients. The other five articles dealt with the analysis of multimorbidity clusters. No article defined big data analytics explicitly.

**Conclusions:**

Although the usage of the phrase “Big Data” is growing rapidly, there is nearly no practical use case for big data analysis techniques in the treatment of multimorbidity in general practice yet. Furthermore, in publications addressing big data analytics, the term is rarely defined.

However, possible models and algorithms to address multimorbidity in the future are already published.

**Electronic supplementary material:**

The online version of this article (10.1186/s12875-019-0928-5) contains supplementary material, which is available to authorized users.

## Background

Patients with more than one chronic disease are commonly defined as multimorbid [1]. Multimorbidity is a highly prevalent condition. In fact, in the United States of America 48% of patients older than 65 years, suffer from more than three chronic diseases. This population accounts for 89% of the annual Medicare’s budget in the USA [2]. When multimorbidity is defined as suffering from at least three chronic diseases, 62% of German patients in general practice older than 65 years are multimorbid [3]. Therefore, multimorbid patients are prevalent in general practice.

Treating the individual diseases of multimorbid patients in accordance with the specific guidelines for the single disease is the most common way to deliver care [4]. This approach carries the danger of leading to an overall deterioration of the health status of multimorbid patients [5, 6]. These challenges intensify with the number of diseases to treat [7].

Limitations for treating these patients described back in 2005 such as drug interactions or guideline related recommendations that contradict each other are still relevant today [8]. To date very few guidelines primarily focus on multimorbidity [9–11]. Therefore, optimization of care for this population is a high-priority task for health care. Currently, new recommendations for improved treatment of multimorbid patients using ehealth, e.g. decision support systems are being published [6, 12].

The term ehealth describes the general use of electronic devices or systems in medical care. One aspect of ehealth is the application of big data analysis techniques. The term “Big Data” was introduced in 1997 [13]. Commonly big data analytics are defined by the “3Vs”: increasing *volume* of data, the high *velocity* of data, and the *variety* of data [14–16]. It is hypothesized that big data analytics have the ability to reveal patterns in patient data that could not be identified with more traditional methods of data analysis [17].

However, there are no cut off values that clearly determine the point at which data starts being big. Still, big data analytics might have the potential to be a useful addition to the treatment of multimorbid patients.

The aim of this review was to evaluate to what degree the application of big data analytics could assist general practitioners in treating multimorbid patients.

## Methods

### Search strategy

Two of the authors (AW, DW) conducted an organized computerized literature search for studies that utilized big data analytics of patient data in order to treat multimorbid patients. This review followed the guidelines of the PRISMA Extension for Scoping Reviews (PRIMSA-ScR) [18].

In January 2018, the databases PubMed, The Cochrane Library, and Web of Science were searched. In order to detect all areas of Big Data Analytics, a complex search strategy was developed, using the terms “big data”, “health analytics”, “healthcare informatics”, “electronic health records”, “databases”, “data collection system”, “electronic data capture”, “data management system”, “deep learning”, “electronic medical record”, “machine learning”, “medical data”, “huge data”, “electronic patient record”, “datamining”, “data analysis”, “reinforcement learning”, “decision support system”, “predictive analytics”, “reasoning” and “inference”. In order to identify studies dealing with big data analytics in the context of multimorbidity, search terms were combined with the terms “multimorb*” and “multi-morb*”.

The terms “general practitioner” or “general practice” were deliberately not included in the search terms to ensure that as many articles as possible on the topic of multimorbidity were included. Relevance to general practice was individually assessed in the screening process.

Additionally, a search of grey literature with Google Scholar was conducted. For this search the terms “multimorbidity AND “big data” AND (“general practice” OR “gp”)” were consented within the research group under the advisement of an expert in computer sciences and used to keep the number of results manageable. Patents and citations were excluded. The search in Google Scholar was performed using the “Private-Setting” in order to produce replicable results.

The results of the searches were imported into the web service Covidence (www.covidence.org) which was used in the further review process. The complete search strategy is available in the supplemental material (Additional file 1).

No review protocol was registered for this scoping review.

### Study screening

After the exclusion of duplicates, 2392 article were included in the review process. Two reviewers (AW, DW) independently screened the titles, abstracts, and subsequently the full-text articles. Discrepancies during the screening process were discussed during regular consensus meetings. A third reviewer (JS) was consulted as needed.

### Eligibility criteria

The authors are members of the “Center for Open Innovation in Connected Health (COPICOH)” at the University of Lübeck. In this center, computer scientists as well as researchers from a variety of health disciplines are working together. The authors held consensus meetings with this research group in order to define, what articles were to be included. It was decided that studies that used standard statistical methods, e.g. large cohort studies that examined data from electronic health records, were not deemed eligible.

After the title and abstract screening, 29 full-texts were screened. Of those full-texts 23 articles were excluded after discussion within the research team because they either did not conduct big data analytics as defined by the authors, did not focus on multimorbidity or were not relevant for general practice.

**Fig. 1:**
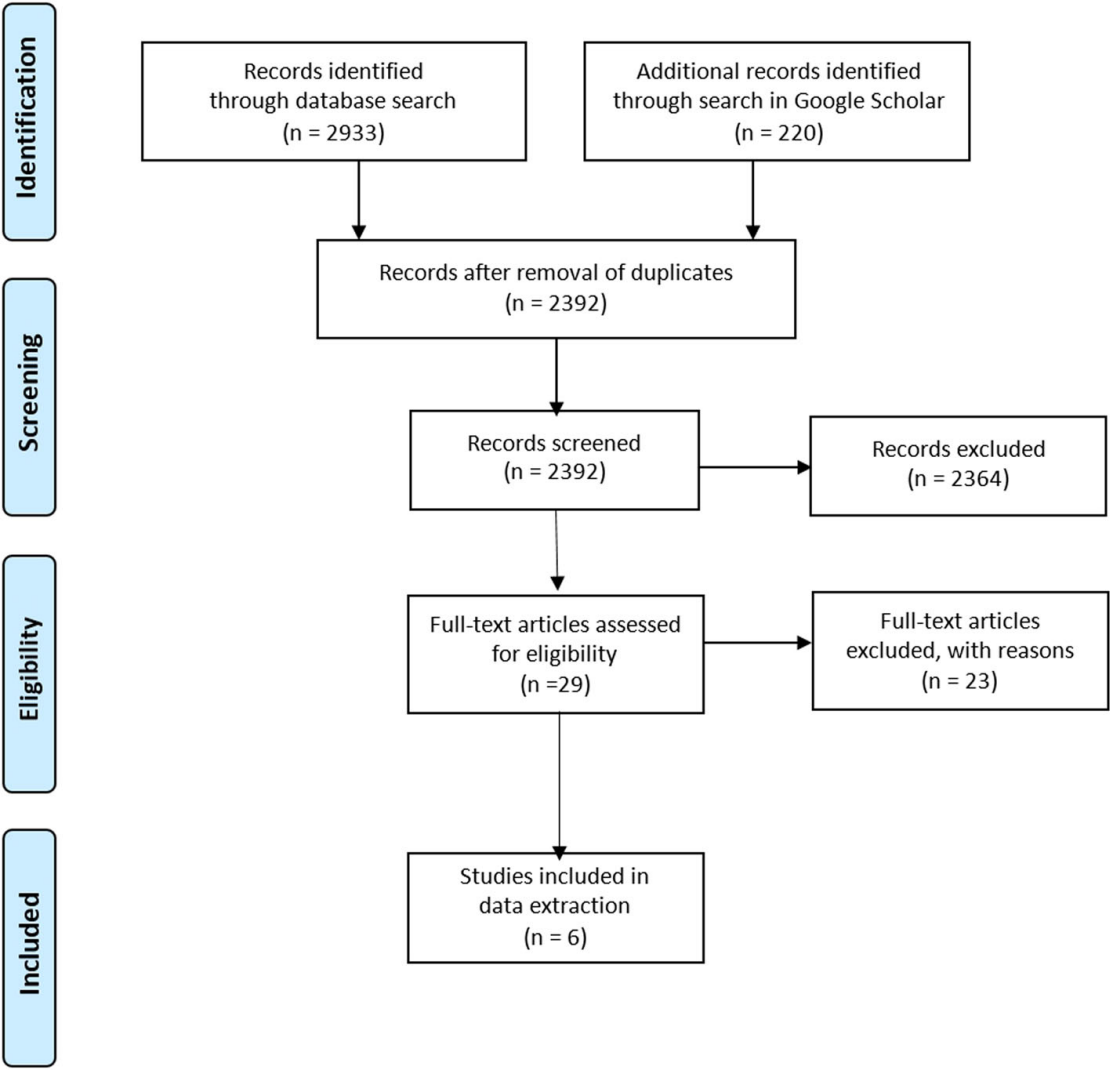
PRISMA Flow Chart

Finally, six articles were included in the data extraction. For better traceability, the entire screening process is visualized using the PRISMA Flow Chart. (see Fig. 1).

### Data extraction

Studies were not filtered by type of study, publication year or language. Validity of studies was evaluated based on the judgement of two independent researchers (AW, DW). The data extraction from the included studies was done by AW and a scientific researcher in the field of computer sciences and consented with all authors.

Extracted data was the publication year, the country of origin, the aim of the study, the number of examined datasets, the used method of data analyses, and the outcome. The Results of the data extraction are summarized in Table 1.

**Table 1:** Summarized characteristics of included studies

Authors	Year	Country	Aims	No. of used observations	Method of data analysis	Outcome
Andriopoulou F, et al. [19]	2013	Greece	Managing patients suffering from chronic conditions.	30	Random Forest	Framework that identifies the necessity to deliver personalized health services by specialists when they are most appropriate.
Schäfer I, et al. [20]	2014	Germany	Depicting which diseases are associated with each other on person-level in multimorbid patients and which ones are responsible for the overlapping of multimorbidity clusters.	98.619 (72.548 for replication analyses)	Analysis based on clustering techniques	Model for the association of diseases to each other. Identification of diseases that form a multimorbidity cluster as well as the identification of diseases responsible for overlapping multimorbidity clusters.
Marx P, et al. [21]	2015	Hungary	Investigating a systems-based approach for the use of separated large-scale multimorbidity data to explore common latent factors of related diseases.	117.803 (subset of the UK Biobank)	MCMC on a Bayesian network	Bayesian, multivariate, system-based approach to identify shared latent factors that could cause multi-morbid diseases without interpreting these factors.
Boshuizen HC, et al. [22]	2017	Netherlands	Determining the magnitude of the difference in the burden of a risk factor with different calculation methods.	Not defined. Study based on the Global Burden of disease database.	Temporal counterfactual reasoning	Dynamic modelling with the DYNAMO-HIA Method estimates the gain in Disability Adjusted Life Years (DALYs) obtained by eliminating exposure to a risk factor more accurately than other established methods.
Kalgotra P, et al. [23]	2017	USA	Addressing the co-occurrences of diseases using network analysis while putting a special focus in disparities by gender.	22.1 million	Network analysis	Identification of different multimorbidity clusters for male and female patients with a prevalence of higher comorbidities in females than males.
Nicholson K, et al. [24]	2017	Canada	Development of the Multimorbidity Cluster Analysis Tool and Toolkit to identify distinct clusters within patients living with multimorbidity.	75.000	Analysis based on clustering techniques	Downloadable Toolkit for analysis and description of combination and permutation of diseases in large datasets of multimorbid patients.

## Results

Of the 220 results in the “grey literature”, none were deemed eligible and consequently were not among the included articles. All of the included articles were published in English. None of these articles used the keyword or term “Big Data”. However, after discussing this within the researcher group, six papers were included. They originated from Greece, Germany, Hungary, the Netherlands, the United States, and Canada [19–24]. The oldest article was published 2013 the latest in 2017.

Four of the articles dealt with the analysis of large data sets of multimorbid patients to analyze or find patterns in the combinations of diseases in these samples [20, 21, 23, 24]. Although the main objectives of the articles may sound similar, the specific focus of each of these articles was a different one. One article proposed a framework for the management of treatment for multimorbid patients who suffered from COPD [19]. The sixth article presented a new dynamic modelling approach to predict the gain in Disability Adjusted Life Years obtained by eliminating exposure to a risk factor more precisely than other models [22].

Another result of this review was that there were no precise definitions for “Big Data” in the screened articles. Furthermore, there were no defined cut-off values to specify at which point the levels of volume, velocity or variety of data are sufficiently high to clearly define them as “Big”.

## Discussion

The aim of this review was to evaluate to what degree big data analytics are already supporting general practitioners in treating multimorbid patients.

Altogether, we identified only one article addressing the approach of improving the treatment of multimorbid patients with COPD by using techniques that are related to big data analytics [19]. However, the approach proposed in this paper has to be further validated including more patients and a broader variety of diseases.

These limitations are in line with other reviews that addressed the benefits of big data analytics for Diabetes type 1 and 2 and Alzheimer’s disease [25, 26].

The other five included articles did not present direct recommendations for the treatment of multimorbid patients. However, they utilized methods and techniques to develop models that could, upon further investigation, shed a better light on the understanding of the underlying patterns for multimorbidity. It would be reasonable to verify these models for the analysis of multimorbidity clusters of other datasets to further the understanding of multimorbidity and then integrate these models into the medical decision framework for treating patients. This missing step of integration is also commonly found in reviews addressing big data analytics in health care [27–29].

Furthermore, we found a lack of clear definitions for the “3 V’s” or the term “Big Data” in general. In other reviews on big data analytics in health care settings, these terms are also only implicitly described, but their definition is usually not included [27, 28].

Although common expectations are, that big data analytics will have a great variety of applications in the field of multimorbid patient treatment in the future [30], this review that found only one study that has direct implications for treatment puts this portrayal in perspective.

These findings might suggest an interface problem between different scientific disciplines that do research in the field of big data. For the clinician finding reliable evidence for the benefit of applying big data analytics to improve treatment is crucial. However, they usually do not have the competence in validating algorithms. Computer scientists may be more interested in developing algorithms for a more generic problem than to apply an algorithm in a specific clinical setting. Therefore, there may be the need for an academic discipline that focuses on the implementation of algorithms into practice. One example of a future application for big data analytics in health care could be the implementation of big data algorithms into medical apps for mobile devices. There are already a number of studies that investigate the possible benefits of these apps [31].

### Strengths and limitations

This is to our knowledge the first review that addresses applying big data analytics in the treatment of multimorbid patients in general practice. Journals commonly included in databases used by health care professionals might not be the ones researcher working in the field of big data analytics are publishing their results in, leading to a bias in our findings.

## Conclusions

One study was found that presents an approach for treating a group of multimorbid patients using big data techniques. Terms pertaining to big data analytics are not defined in studies applying these methods. Over all, there seems to be a mismatch between the perceived presence and usage of big data in health care and existing literature in databases commonly used by health care professionals. It seems highly relevant to form interdisciplinary research environments in which experts in implementing computer sciences and health care professionals work together to evaluate the benefits of big data analysis techniques for the treatment of patients.

## Additional file

Additional file 1: Search Strategy.pdf. Detailed Information on search strategy. Information on search strategy, search results, and exclusion criterias. (PDF 47 kb)

## Abbreviations

COPD: Chronic obstructive pulmonary disease
COPICOH: Center for Open Innovation in Connected Health
PRISMA: Preferred Reporting Items for Systematic reviews and Meta-Analyses
PRISMA-ScR: PRISMA Extension for Scoping Reviews
USA: United States of America
