# Supplementary material for: Are big data analytics helpful in caring for multimorbid patients in general practice? - A scoping review
Source: BMC Fam Pract. 2019 Feb 27;20:37. doi: 10.1186/s12875-019-0928-5 (PMC6394098; doi:10.1186/s12875-019-0928-5)
Supplement: Supplementary file 1 — Search Strategy.pdf. Detailed Information on search strategy. Information on search strategy, search results, and exclusion criterias. (PDF 47 kb) [file 12875_2019_928_MOESM1_ESM.pdf]

Additional File 1: Detailed Information on search strategy

a) Search Strategy

Big data	multimorb*
Health analytics	multi-morb*
Healthcare informatics	
Electronic health records	
Databases	
data collection system	
electronic data capture	
data management system	
deep learning	
electronic medical record	
machine learning	
medical data	
huge data	
electronic patient record	
datamining	
data analysis	
reinforcement learning	
decision support system	
predictive analytics	
reasoning	
Inference	

Search term:

((((((((((((((((((((Big data) OR Health analytics) OR Healthcare informatics) OR Electronic health records) OR Databases) OR data collection system) OR electronic data capture) OR data management system) OR deep learning) OR electronic medical record) OR machine learning) OR medical data) OR huge data) OR electronic patient record) OR datamining) OR data analysis) OR reinforcement learning) OR decision support system) OR predictive analytics) OR reasoning) OR Inference) AND ((multimorb*) OR multi-morb*)

b) Detailed Information about the search results

PubMed	1.701 hits
Web of Science	1.162 hits
Cochrane Library	70 hits
Grey Literature	220 hits
**Total:**	**3.153 hits**
Removed duplicates	761
**Screened articles**	**2.392**

Date of most recent search executed: 29th of August 2018

c) Table of exclusion criteria for full texts

Not big data as predefined	14
Focus of article was not the use of big data in the treatment of multimorbidity	5
Full Text was not retrievable	2
Focus of article was not the treatment of multimorbidity	1
**Total number of excluded full texts**	**22**
